# Affective Neuroscience of Loneliness: Potential Mechanisms underlying the Association between Perceived Social Isolation, Health, and Well-Being

**DOI:** 10.20900/jpbs.20220011

**Published:** 2022-12-26

**Authors:** Anna J. Finley, Stacey M. Schaefer

**Affiliations:** Institute on Aging, University of Wisconsin-Madison, Madison, WI 53706, USA

**Keywords:** loneliness, perceived social isolation, affective neuroscience, depression, well-being, mental health, physical health, social determinant of health

## Abstract

Loneliness, or the *subjective feeling* of social isolation, is an important social determinant of health. Loneliness is associated with poor physical health, including higher rates of cardiovascular disease and dementia, faster cognitive decline, and increased risk of mortality, as well as disruptions in mental health, including higher levels of depression, anxiety, and negative affect. Theoretical accounts suggest loneliness is a complex cognitive and emotional state characterized by increased levels of inflammation and affective disruptions. This review examines affective neuroscience research on social isolation in animals and loneliness in humans to better understand the relationship between *perceptions* of social isolation and the brain. Loneliness associated increases in inflammation and neural changes consistent with increased sensitivity to social threat and disrupted emotion regulation suggest interventions targeting maladaptive social cognitions may be especially effective. Work in animal models suggests the neural changes associated with social isolation may be reversible. Therefore, ameliorating loneliness may be an actionable social determinant of health target. However, more research is needed to understand how loneliness impacts healthy aging, explore the role of inflammation as a potential mechanism in humans, and determine the best time to deliver interventions to improve physical health, mental health, and well-being across a diverse array of populations.

## INTRODUCTION

Humans are social animals with intricate social networks and complex social cognition. We suffer acute stress from *perceived* social isolation—commonly referred to as loneliness. Loneliness is related to but distinct from objective measures of social isolation (i.e., frequency of social contact), as subjective and objective measures of social isolation are only weakly correlated at about *r* = 0.20 [1]. Evidence is mounting that loneliness is a complex affective state that cannot simply be remedied by increasing frequency of social contact [2–4]. Loneliness impacts an estimated 25%–50% of the US population at a given time [3,5], and older adults may be particularly at risk—1 out of 3 individuals over age 45 report feeling lonely, rising to 1 out of 2 individuals over 45 with low income [6]. Critically, loneliness is recognized as a social determinant of health [7–10], associated with greater risk for cardiovascular disease [11–13], dementia and cognitive decline [14–23], the development and exacerbation of mood disorders including depression [2,24–29], and increases in mortality comparable with smoking [26,30–33]. Eliminating loneliness could prevent an estimated 11%–18% of cases in depression in individuals over 50 [2]. Given the devastating associations between loneliness and health, affective neuroscientists are working to better understand the lonely brain and its emotional processes. This review outlines recent work regarding the associations of loneliness with emotions, health, and well-being, followed by recent affective neuroscience findings regarding associations between loneliness and the brain in socially isolated animals (as a proxy for perceptions of social isolation) and lonely humans. We conclude with a discussion of potential intervention targets and suggestions for future research.

## THEORIES OF LONELINESS

The most established theoretical explanation of loneliness is John Cacioppo’s Evolutionary Theory of Loneliness [3]. Cacioppo’s theory posits that loneliness initiates a highly conserved biological response that, while adaptive in the short-term when faced with a lack of social contact, is physically and psychologically maladaptive in the long-term [3]. Although the response to loneliness resembles the biological response to other chronic stressors, the perceived social context is posited to additionally trigger an affective bias focused on self-preservation, with enhanced sensitivity to social threat and increased motivation to restore social connection. This bias is theorized to result in a vicious cycle stemming from dysregulated affective responding, whereby lonely individuals are more likely to interpret ambiguous social information negatively, resulting in behaviors and cognitions that undermine social connections and increase feelings of loneliness [3,34].

Although not solely developed to describe loneliness, George Slavich’s Social Safety Theory provides another theoretical framework for the human response to loneliness. Slavich’s theory posits that conditions of social threat, including subjective perceptions of isolation, trigger a specific immune response tuned to prepare for physical injuries (more likely to happen when a social animal is isolated from a social group) and reduce preparedness for viral infections (less likely to happen when isolated from a social group) [35,36]. If chronic, this increase in inflammation has been linked to an increase in a wide range of affective disruptions as well as mental and physical disorders [35].

Both theories posit that changes in affective processes underlie many of the negative effects of loneliness, particularly because abnormal or context-inappropriate emotional responses can impair functioning and increase vulnerability to psychopathology [37,38]. Behavioral studies indicate lonely individuals are more vigilant towards (social) threat [25,39–42], including increased attention to images of social rejection and threat as measured by eye tracking [43,44]; more likely to mislabel emotional expressions as negative [45]; and faster to identify negative emotional faces (including angry [46], sad, and fearful faces [47]). Additionally, loneliness is associated with poorer sleep quality [48,49]; increases in depression and negative affect [5,24,50,51]; increased activation of the hypothalamus-pituitary-adrenal (HPA) axis, which plays a role in the body’s response to stress (particularly through increased release of glucocorticoids including cortisol [11,52,53]); and increased circulating levels of pro-inflammatory cytokines and inflammatory compounds (e.g., interleukin-6 (IL-6), C-reactive protein, and fibrinogen [11,54–58]). Inflammation may be an important mechanism linking the disrupted affective processes associated with loneliness to negative health outcomes, such as cardiovascular disease [11–13], as well as the greater risk of long COVID associated with loneliness [59]. However, the relationship between loneliness and inflammation is likely bidirectional [60,61], as drug-induced inflammation temporarily increases feelings of social disconnection in humans [55]. Yet, research linking the negative mental and physical health outcomes associated with loneliness to the underlying inflammatory mechanistic processes is still needed to better understand how loneliness “gets under the skin” and negatively impacts health and well-being, and to identify the mechanisms to target to reduce the adverse impacts of loneliness.

## INFLAMMATION, AFFECT, AND THE BRAIN

Higher levels of inflammation are associated with affective dysregulation: inflammation increases neural sensitivity to threat [62] and precipitates “sick” behaviors including fatigue, low activity, and depressed mood [60]. Depression is often comorbid with inflammatory diseases such as asthma, rheumatoid arthritis, inflammatory bowel disease, and heart disease [63]; individuals with depression have higher levels of pro-inflammatory cytokines [64]; and administration of drugs and vaccines that induce an inflammatory reaction increase reports of depressive symptoms and negative mood [55,60]. In animal models, inflammation induces depressive-like behaviors (i.e., sleep disturbances, reduced sucrose consumption suggesting anhedonia), and decreases expression of neuroprotective hormones in the hippocampus and medial prefrontal cortex [65,66]. An extensive review of the influence of pro-inflammatory cytokines on the brain is beyond the scope of this review, but multiple, often bidirectional, biological pathways exist for inflammation to influence regions associated with stress and emotion, including the anterior insula, amygdala, and hippocampus [60,61,63].

## NON-HUMAN ANIMAL MODELS OF SOCIAL ISOLATION

In animal models, causation can be assessed by experimentally assigning animals to be housed in social isolation. Still, social isolation in animals is only a proxy for human loneliness without self-reported *perceptions* or feelings [53]. Research in bumblebees [67] to rodents and monkeys [68] show social isolation results in profound biological, behavioral, and neural changes. In particular, isolating social mammals increases systemic glucocorticoid levels and pro-inflammatory cytokines [34,69], and changes behavior (i.e., reduced exploration, increased fear response), such that social isolation is used as an animal model of mood disorders [34,69–71].

Social isolation in rodents and non-human primates results in reductions in cellular proliferation, neurogenesis, neuroplasticity, and myelination in the hippocampus [72,73], amygdala [68], and prefrontal cortex (PFC) [71,74,75]. The changes in these brain structures may contribute to affective dysregulation given the role of the hippocampus in learning, memory, emotion, and regulation of the HPA axis [76]; the association of the amygdala with affective responses, including responding to salient and arousing information and events, as well as social behavior [77,78]; and the PFC’s involvement in regulating stress and affective states [79]. Promisingly, findings in rodents suggest that the impacts of social isolation on the brain are reversable. Resocialization has been found to improve memory and reduce anxious and depressive behavior [80], reverse neuronal restructuring in the hippocampus [72,81], normalize gene expression related to neuroplasticity in the amygdala [82], and reverse changes in the PFC [83]. Although currently limited primarily to rodents, these encouraging findings highlight the importance of developing and testing effective loneliness-reducing interventions in humans to learn whether loneliness-related neural changes are reversible in humans.

The animal findings are consistent with both the associations between loneliness and affective disruptions in humans, as well as known impacts of inflammation on the brain. However, while animal models provide experimental control, how closely the animal findings describe the mechanisms in humans is unknown given the difficulty identifying homologous neural structures across species [79] and the inability to assess the subjective feeling of social isolation in non-human animals [53].

## AFFECTIVE NEUROSCIENCE OF LONELINESS IN HUMANS

Unfortunately, many human neuroscience studies fail to distinguish between measures of social integration and *objective* social isolation (such as social network size, number of social interaction partners, frequency of social interactions) with perceptions of *subjective* social isolation (loneliness, unmet social needs, feeling socially disconnected from others). We focus on the limited studies of subjective feelings of social isolation (i.e., loneliness) and associated differences in brain structure and function.

Consistent with behavioral associations of loneliness and altered attention to and perceptions of (social) threats ([84] for a review), task-based neuroscience research using electroencephalography (EEG; measures electrical changes from firing neurons) and functional magnetic resonance imaging (fMRI; measures blood oxygenation changes in the brain) show differences in affective neural responses with loneliness. Lonely individuals more quickly differentiate negative social words compared to non-social negative words [39], as well as threatening social images compared to non-social threatening images [40] as assessed by EEG microstates. Lonely individuals have a faster neural response measured with EEG event related potentials to emotional faces (as indicated by a faster N170 component), as well as an attentional bias towards negative faces (as indicated by a larger P100 component) [85]. Positive affective responses to social cues may also be disrupted in loneliness. Lonely relative to nonlonely individuals show less fMRI activity to positive social images of strangers in the ventral striatum [86] but greater ventral striatum activity when viewing images of a close other [87].

Loneliness appears to disrupt neural activity related to social behavior and cognitive control. Lonely individuals report less interpersonal trust, act in a less trusting manner, and show decreased fMRI activity in brain regions important for emotional processes, such as the amygdala and nucleus accumbens, during an interpersonal trust game compared to a risk game [88]. In a behavioral synchronization task, lonely individuals show increased fMRI activity in inferior frontal gyrus and inferior parietal lobule (associated with mirroring behaviors) and worse behavioral synchronization with a partner compared to non-lonely individuals [89]. Finally, in the only task-based neuroscience study to focus on older adults (aged 61–75), loneliness with high levels of depression symptoms was associated with decreases in inhibitory control (as indicated by a smaller P300 measured with EEG during a go/no-go task) [90], suggesting older individuals who are lonely and depressed may experience the most disrupted cognitive function.

These task-based neuroscience studies suggest profound neurofunctional differences in how lonely individuals perceive social threat and social partners, as well as disrupted cognitive control. Disrupted perceptions and cognitions may lead to a vicious cycle characterized by increased negative affect, decreased trust of others, and reductions in affiliative social behavior, consistent with theoretical accounts of loneliness [3,35]. However, research directly connecting these changes in neural responding and social behavior outside of lab-based tasks is lacking.

Differences in neural activity at rest associated with loneliness have been examined using resting state functional connectivity analyses of fMRI data, which assesses how networks of brain regions are coactivated. This work has associated loneliness with connectivity shifts in the default network (DN; associated with mentalizing and daydreaming), and the limbic network (implicated in emotional processes) [91]. Lonely individuals show increased DN activity [91,92] and less interconnections between the DN and other brain networks [93]. Additionally, loneliness is associated with increased activity in networks associated with monitoring the external environment for salient features, including the ventral attention network (VAN) [93–97]. A whole-brain machine learning model predicted loneliness from resting state functional connectivity data, with the connections within and between networks important for cognitive control, emotional processing, and social perception being most predictive [98]. A functional connectivity meta-analysis examining differences in task-related connectivity with loneliness found altered patterns in the insula, superior frontal gyrus, medial frontal gyrus, and the striatum, which overlapped with the VAN, frontal parietal network (implicated in higher cognitive processes) and DN [99]. The authors suggest the results indicate that loneliness is associated with increased mentalizing, perhaps to fill the social void or ruminate over negative social interactions, as well as an increase in vigilance towards external threats [91,92,100]. However, this remains speculative as loneliness-associated shifts in functional connectivity have not yet been directly related to increases in vigilance, mentalizing, or rumination.

Neuroanatomical differences associated with loneliness have been assessed with structural MRI and diffusion tensor imaging (DTI; an MRI technique, assesses white matter density and integrity). Consistent with findings in non-human animals, lonely individuals show reduced volume in the PFC (particularly medial and dorsolateral regions), amygdala, hippocampus, and ventral striatum [97,101,102]. Reduced volume associated with loneliness has also been reported in the insula [97,102] (associated with social processes and self-awareness as well as integrating affective, cognitive, visual, and sensorimotor networks [103]), and the posterior temporal cortex (including the temporoparietal junction and posterior superior temporal sulcus; associated with social cognition [97,102,104]). Lonely individuals show poorer myelination generally [105,106] and in areas of the PFC, insula, and the posterior temporoparietal junction [97,107], but greater microstructural integrity of the fornix, the major output white matter tract of the hippocampus, implicated in emotional processing, memory, and mentalizing [91]. Additionally, hippocampal volume and volume of DN nodes have been found to covary significantly in loneliness, and this relationship is further associated with greater fornix tract white matter integrity [92]. Loneliness has been linked with accelerated aging, such that loneliness is associated with larger gaps between chronological age and an estimate of brain age based on structural MRI data (i.e., lonely individuals have “older” brains than expected) [108]. Overall, the structural and functional differences with loneliness are consistent with altered affective perceptions and regulation, disruptions in cognitive control, and hint that loneliness is associated with accelerated neural aging.

Taken together, the existing neuroscientific research on loneliness suggests that *perceived* social isolation has profound impacts on the brain relating to emotion, social perception, cognitive function, and aging. These findings underly the importance of reducing loneliness, and provide insights into the mechanism behind the links between loneliness and poor mental health and cognitive decline. In particular, dysregulated emotions and altered perceptions of social interactions, in combination with altered resting state networks consistent with increased vigilance or rumination may lead to the development or exacerbation of mood disorders. The association between loneliness and smaller hippocampal volumes and accelerated aging suggests that loneliness may be a particular potent factor in the development or exacerbation of cognitive decline and dementia, but current research on the role of loneliness in unhealthy aging is too heterogeneous to draw strong conclusions [22]. Future research on loneliness should consider assessing additional biomarker outcomes to gain a deeper understanding of the relationship between loneliness, health, and well-being throughout the life course and the biological mechanisms that may underly how loneliness contributes to ill health and faster brain aging.

## PROMISING INTERVENTION TARGETS

The literature reviewed above suggests widespread affective disruptions are implicated in the negative outcomes associated with loneliness. In particular, greater attention to (social) threat and disruptions in affective appraisals, responding, and regulation as demonstrated behaviorally and neurally suggests that interventions should target social and emotional perceptions and appraisals. Indeed, a meta-analysis on anti-loneliness interventions found that interventions that addressed maladaptive social cognitions reduced feelings of loneliness more than interventions focused on improving social skills, social support, or opportunities for social interaction [109]. A randomized control trial with homebound older adults found a behavioral activation intervention [110] designed to identify goals for social connectedness and strategize how to overcome obstacles (including maladaptive social cognitions) reduced depression and disability symptoms, increased social connection, and decreased loneliness compared to weekly social video calls [111]. Thus, interventions that target dysregulated social emotions, cognitions, and behaviors associated with loneliness may be particularly effective in reducing loneliness. Unfortunately, no research has assessed if interventions normalize disrupted neural patterns or assessed other biomarkers [112]. Future intervention work should address maladaptive social and cognitive processes and include a wide range of outcome measures, including neural and health measures, to holistically assess efficacy and inform clinicians and policy makers on how to reduce the negative health and well-being outcomes associated with loneliness. This may be particularly important to understand how different interventions are impacting individuals and assessing who may benefit the most from a particular intervention type. Furthermore, interventions that reduce self-reported loneliness but do not address the underlying mechanisms causing poor health and well-being outcomes will not be effective health interventions.

## FUTURE RESEARCH

Future research needs to directly connect loneliness-associated differences in the brain with social and affective dysregulation in daily life. The causal role of inflammation and increased HPA activation also needs additional careful investigation. While non-human animal models find social isolation leads to increased inflammation, work in humans has found inflammation increases feelings of social isolation [55], suggesting complex bidirectional relationship between inflammation and loneliness. The question remains, are the neural and inflammatory differences associated with perceptions of social isolation caused by loneliness, or do they predispose individuals to feel lonely? Understanding the directionality of this relationship will be critical to understanding what interventions will be most beneficial for improving health and well-being.

Given the vulnerability of older adults to loneliness [6] and the association between loneliness and cognitive decline and dementia [14–20], a surprising lack of studies focus on the lonely older brain ([90,101,108] are notable exceptions). While many of the mechanisms of loneliness are likely similar across the lifespan, future longitudinal work should focus on a wide age-range of children and adults featuring children, adolescents, young adults, middle-aged adults, and older adults to determine how episodes of loneliness impacts developmental and aging processes at different stages of life, with a focus on identifying critical periods to intervene and different intervention approaches depending on life stage. Additionally, the causal relationship between loneliness and cognitive decline is unclear [22]. Does loneliness have a causal impact on accelerated cognitive decline, or are individuals who are experiencing cognitive decline more likely to be lonely? Can anti-loneliness interventions slow cognitive decline and reduce rates of dementia? Given the devastating course of cognitive decline/dementia and projections for large increases in their incidence and associated health-care costs [113,114], the evidence linking smaller hippocampal volumes and accelerated metrics of brain aging with loneliness suggests interventions to reduce loneliness are critically needed to evaluate their potential to slow cognitive decline for some individuals.

Most research does not differentiate between transient and chronic feelings of loneliness. However, the length of time an individual feels lonely may have important implications. For instance, while individuals with chronic loneliness measured over multiple timepoints had increased risk of Alzheimer’s disease compared to individuals who never reported feeling lonely, transient loneliness (i.e., loneliness measured at one longitudinal timepoint that was resolved by the next assessment) was associated with reduced risk of Alzheimer’s disease [19]. More work is needed to determine the impact of different lengths of loneliness episodes on the brain, health, and well-being, at different life stages, and identify the most critical times to intervene.

Research also needs to explicitly study a wide range of communities that may be differentially impacted by loneliness. While loneliness is a ubiquitous human phenomenon, how it may manifest and what interventions are most effective may differ by culture or social status. For example, loneliness mediates the relationship between gendered racism and symptoms of depression and anxiety in Black women [115]. Additionally, loneliness is more common among adolescents and young adults, the elderly, BIPOC individuals, people who identify as LGBTQ, as well as individuals with low socioeconomic status, chronic health conditions, and/or disabilities, and among people who live in rural or under-resourced areas [6,112,116,117]. Future work needs to examine how loneliness differentially impacts different populations and how best to address loneliness within those communities.

## CONCLUSIONS

Affective neuroscience research on loneliness has associated subjective feelings of social isolation with an array of biological and neural changes consistent with altered social, affective, and cognitive processes. Theoretical accounts of loneliness suggest these changes can lead to a vicious cycle whereby loneliness is exacerbated and well-being and health are reduced. Animal studies suggest that these neural changes may be reversible, while human interventions targeting the maladaptive social cognitions associated with loneliness are so far the most effective at reducing perceptions of social isolation. Ameliorating loneliness holds promise as an actionable social determinant of health for policy makers and healthcare providers to target to reduce incidents of mood disorders and cognitive decline. Further research is needed to understand the causal direction of these neural differences, the role of inflammation as a potential mechanism, and identify interventions that change both self-reports of loneliness as well as biological and neural markers of dysregulated affective processes throughout the lifespan to ameliorate both the pain of feeling lonely and the detrimental impacts feeling lonely has on the body and brain.

## Data Availability

Data sharing not applicable to this article as no datasets were generated or analyzed during the current study.

## ABBREVIATIONS

HPA: hypothalamus-pituitary-adrenal
IL-6: interleukin-6
PFC: prefrontal cortex
EEG: electroencephalography
fMRI: functional magnetic resonance imaging
DN: default network
VAN: ventral attention network
DTI: diffuse tensor imaging
BIPOC: Black, Indigenous, and people of color
LGBTQ: lesbian, gay, bisexual, transgender, and questioning
